# Mapping global evidence on compassion fatigue among healthcare workers during COVID-19: insights and implications for future preparedness – a scoping review

**DOI:** 10.7189/jogh.16.04130

**Published:** 2026-04-17

**Authors:** Laston Gonah, Themba G Ginindza, Khumbulani Welcome Hlongwana

**Affiliations:** 1 School of Public Health, Faculty of Medicine and Health Sciences, Walter Sisulu University, Mthatha, South Africa; 2 Discipline of Public Health Medicine, School of Medicine, College of Health Sciences, University of KwaZulu-Natal, Durban, South Africa; 3 Cancer & Infectious Diseases Epidemiology Research Unit (CIDERU), College of Health Sciences, University of KwaZulu-Natal, Durban, South Africa

## Abstract

**Background:**

Compassion fatigue (CF) is a critical occupational hazard for healthcare workers (HCWs), intensified by the COVID-19 pandemic, with implications for well-being, retention, and quality of care. We aimed to map the global evidence on CF prevalence, risk factors, effects, interventions, and research gaps among HCWs during the COVID-19 pandemic.

**Methods:**

A scoping review of 56 studies from 21 countries (2020–2025) was conducted following PRISMA-ScR guidelines. Seven databases were searched, and findings were synthesised narratively with attention to occupational, demographic, and systemic determinants of CF.

**Results:**

Compassion fatigue prevalence ranged from 20 to 87%. It was most pronounced among nurses, women, frontline staff, early-career professionals, and those in under-resourced or rural settings. Key risk factors included high workload, long shifts, repeated exposure to death, moral distress, and limited organisational support. Symptoms encompassed emotional exhaustion, depersonalisation, diminished empathy, and co-occurring anxiety, depression, or secondary traumatic stress. Interventions (resilience and peer-support programmes, self-compassion training, motivational messaging, and mobile psychoeducation) showed small-to-moderate benefits but were limited by methodological heterogeneity and scarce robust evaluation. Temporally, CF peaked during early pandemic surges and persisted among frontline staff and in resource-constrained or long-COVID contexts.

**Conclusions:**

Compassion fatigue is a multifactorial, context-dependent hazard disproportionately affecting vulnerable HCWs. Effective mitigation requires longitudinal research, inclusive global representation, and multi-level strategies linking individual resilience with organisational reform and policy action to safeguard HCW well-being in current and future crises.

Globally, the COVID-19 pandemic placed unprecedented psychological demands on healthcare workers (HCWs), exposing them to prolonged suffering, moral distress, and resource scarcity [1,2]. These pressures predisposed HCWs to developing compassion fatigue (CF), a state of emotional, physical and psychological exhaustion arising from sustained caregiving burdens [3]. While much attention has focussed on patient outcomes and population mental health, the well-being of HCWs, which is critical to sustaining healthcare systems, has often been overlooked [1,4].

Compassion fatigue threatens workforce productivity, manifesting through reduced empathy, irritability, burnout, and compromised quality of care [5, 6]. Recognising HCWs as a vulnerable subgroup is essential for building resilient health systems and informing future pandemic preparedness [7]. However, evidence on CF during COVID-19 remains fragmented, with unclear patterns across populations, settings, and interventions, thereby limiting actionable insights for policy and practice.

This scoping review maps global literature on CF among HCWs during COVID-19, examining prevalence, risk factors, interventions, and research gaps. By identifying patterns and evidence deficits, the review aims to provide insights that can guide multi-level strategies for workforce resilience and health system preparedness in similar or related future crises.

## METHODS

### Study design

This was a scoping review conducted following the Preferred Reporting Items for Systematic Reviews and Meta-Analyses extension for Scoping Reviews (PRISMA-ScR) framework to ensure transparency, reproducibility, and methodological rigour.

### Search strategy

A comprehensive literature search was conducted in MEDLINE (Ovid), Embase (Ovid), PsycINFO (Ovid), CINAHL (EBSCOhost), Scopus, Web of Science, and Google Scholar to identify studies examining CF among HCWs during the COVID-19 pandemic. The search covered the period from January 2020 to November 2025, with the final search conducted on 7 November 2025, immediately prior to manuscript submission.

Only peer-reviewed articles published in the English language were included. Studies were excluded if they did not investigate a clearly defined study population or failed to report essential methodological characteristics such as sample size, study setting (place) and research design. Preprints, conference papers and review articles were also excluded. The exclusion of these sources was done to ensure that the mapped evidence was based on complete, peer-reviewed primary research with sufficient methodological detail to allow meaningful interpretation, comparison and synthesis of findings. No geographic restrictions were applied.

The search strategy combined terms related to CF, COVID-19, and HCWs, along with synonyms for professional roles, risk factors, coping strategies, interventions and research gaps, and was adapted for each database using appropriate controlled vocabulary and syntax. In MEDLINE (Ovid), the search strategy was: (‘compassion fatigue’ OR ‘secondary traumatic stress’ OR ‘professional quality of life’) AND (‘COVID-19’ OR ‘SARS-CoV-2’ OR ‘coronavirus’) AND (‘health care worker*’ OR nurse* OR physician* OR ‘health personnel’ OR ‘frontline staff’). Equivalent terms and indexing were applied in other databases using Boolean operators, truncation, and field limits to ensure consistency and sensitivity across searches.

Google Scholar was searched using the phrase ‘compassion fatigue AND COVID-19 AND health care workers’. Results were sorted by relevance, and the first 200 records were screened, consistent with established methodological guidance for scoping reviews using Google Scholar [8,9]. Titles and abstracts were assessed for eligibility, and full texts were retrieved where relevance could not be determined from the abstract alone. Reference lists of all included studies were also hand-searched to identify additional eligible articles not captured through database searching.

### Data extraction and synthesis

A structured extraction form captured study characteristics (author, year, country, study design, population, sample size, and measurement tools), key findings (prevalence, risk factors, symptoms/effects, interventions), and identified gaps.

Articles were independently extracted and screened in COVIDENCE by three researchers, with discrepancies resolved through discussion guided by the study’s objectives and scope. Thematic synthesis followed a structured, multi-step process. Three reviewers independently conducted open coding of extracted data in NVivo 14 to identify key patterns in CF, including prevalence, risk factors, effects, interventions, and research gaps. Codes were then grouped into candidate themes through axial coding [10]. Coding consistency was independently verified by two reviewers, with discrepancies resolved through iterative discussion and consensus meetings, ensuring analytic rigor and reliability. Theme development was guided by constant comparison across studies, with analytic memos used to refine theme boundaries and ensure alignment with the review objectives.

## RESULTS

### Article screening process

**Figure 1:**
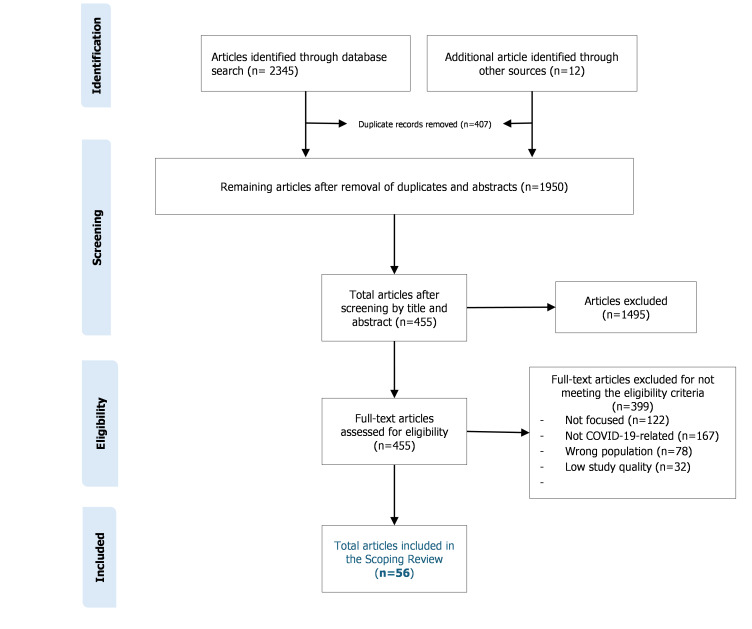
Article screening process using the Preferred Reporting Items for Systematic Reviews and Meta-Analyses extension for Scoping Reviews (PRISMA-ScR) flow diagram [9]. Numbers indicate records identified, screened, excluded, and included at each stage of the review.

The article selection process was conducted in Covidence, guided by the PRISMA statement (**Figure 1**): records identified → screened → excluded → quality-screened → included (n = 56).

Although formal quality appraisal is not mandatory in scoping reviews, a pragmatic methodological screening was applied in this review because of the policy-relevant and practice-sensitive nature of COVID-19 workforce evidence, where inclusion of studies with severe methodological limitations could risk misleading interpretations of prevalence and associated factors. This approach aligns with emerging guidance suggesting that quality screening may be appropriate when scoping reviews aim to inform health system decision-making rather than solely map volume of evidence [8]. The quality screening was not intended to rank evidence or infer effect sizes, but to exclude studies with insufficient methodological transparency to support meaningful synthesis.

An adapted 6-item quality assessment tool for systematic reviews of observational studies (QATSO) [11] tool was applied at the eligibility stage of the PRISMA flow diagram to identify studies with substantial methodological limitations (**Table 1**). This tool evaluates six aspects/items: study sample representativeness; adequacy of sample size; clarity of eligibility criteria; objectivity and appropriateness of outcome measurement; implementation of measures to address bias in measurement or analysis, and control or adjustment for confounding. Each of the six items is scored on a binary scale (Yes = 1, No = 0), resulting in a total score ranging from 0 to 6 for each article. Articles scoring below 3 points were considered of poor quality and excluded from the study. Of the 88 full-text articles assessed for eligibility, 32 were excluded following pragmatic quality screening, resulting in 56 studies included in the final synthesis.

**Table 1:** Scoring of review articles

Grading	Total score (out of 6 points)		
	**5 or 6 points**	**3 or 4 points**	**0, 1, or 2 points**
Risk of bias	Low	Medium	High
Study quality	Good	Satisfactory	Poor
Number of articles	11	45	32
Total included articles scoring ≥3/6: n = 56 (11 + 45)			

### Characteristics of included studies

**Figure 2:**
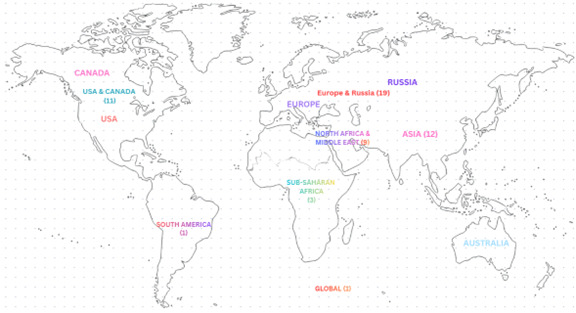
Global spread of selected articles (numbers in brackets indicate the number of articles).

A total of 56 studies, conducted across 21 countries and published since 2020, were included in this scoping review. Most studies originated from Europe (n = 19) and Asia (n = 12), followed by North America (USA and Canada, n = 11) and the North Africa and Middle East region (n = 9). Overall, the global distribution of publications on CF among healthcare workers during the COVID-19 pandemic was uneven, highlighting geographic gaps in the evidence base (**Figure 2**). Evidence from sub-Saharan Africa was limited (n = 3), with only one study each from South America and global multi-region contexts (n = 1).

Most studies (n = 46, 82.1%) employed cross-sectional designs [3–7,12–50], including descriptive and correlational approaches. Fewer studies (n = 5, 8.9%) used mixed-methods designs [2,51–54], four studies used interventional or randomised controlled trial designs with sample sizes ranging from six to 70 participants [1,55–57], and only used qualitative approaches [58]. Nurses were the most frequently studied population (n = 33 articles, 58.9%), followed by multidisciplinary healthcare workers, including physicians (n = 10, 17.9%), intensive care unit and emergency staff (n = 4, 7.1%), mental health professionals (n = 2, 3.6%), nursing students or interns (n = 2, 3.6%), and caregivers or palliative care personnel (n = 2, 3.6%). Three studies included unspecified healthcare worker populations (**Table 2**). Sample sizes varied substantially, ranging from eight participants in small qualitative studies to 2256 participants in large-scale surveys, with an estimated cumulative sample of approximately 22 336 participants across all included studies.

**Table 2:** Summary article characteristics (n = 56)

Article characteristic	Number of articles (n)
**Study design**	
Cross-sectional*	46
Mixed methods	5
RCT/quasi-experimental/interventional	4
Qualitative	1
**Study setting**	
Single setting	39
Multicentre	14
National	2
Multinational/global	1
**Study population**	
Nurses	33
Multidisciplinary HCWs (including physicians)	10
ICU/ED staff	4
Mental health professionals	2
Nursing students/interns	2
Caregivers/palliative	2
Unspecified professions of health care workers	3

HCW – healthcare worker, ICU/ED – intensive care unit/emergency department, RCT – randomised controlled trials

*Including descriptive cross-sectional, cross-sectional online survey, cross-sectional multicentre, comparative cross-sectional, cross-sectional analytical, cross-sectional correlational, cross-sectional mixed-methods quantitative.

Assessment of compassion fatigue among healthcare workers during and after the COVID-19 pandemic was predominated by cross-sectional research [3–7,12–50], with relatively fewer randomised controlled trials (RCTs) [1], quasi-experimental [56], and other interventional studies [55,57] (**Table 3**).

**Table 3:** Summary of study designs and geographic distribution of included studies (n = 56)

Study design category	Countries represented	No. of studies
Cross-sectional*	Turkey, USA, Philippines, India, Serbia, Nepal, Egypt, Italy, Iran, Canada, Nigeria, Romania, China, Spain, Tunisia, Colombia, Uganda, Greece, Saudi Arabia, Japan, South Korea	46
Mixed methods	Turkey, Italy, Canada, USA, Greece	5
RCT/quasi-experimental/interventional	Turkey, USA, Iran	4
Qualitative	Turkey	1

RCT – randomised controlled trials

*Including descriptive cross-sectional, cross-sectional online survey, cross-sectional multicentre, comparative cross-sectional, cross-sectional analytical, cross-sectional correlational, cross-sectional mixed-methods quantitative.

### Measurement tools

Across the 56 studies, CF measurement primarily relied on two validated instruments: the Professional Quality of Life Scale (ProQOL; versions IV and V) and the Compassion Fatigue Short Scale (CF-SS). ProQOL was the most widely used measure (29 studies), assessing CF alongside compassion satisfaction and burnout within the same conceptual framework, followed by CF-SS (17 studies). A small number of studies adapted or combined these scales with context-specific items or qualitative probes (eight studies), but few introduced new or unvalidated tools (two studies). This variability in measurement instruments and conceptual framing of compassion fatigue across studies and contexts could have contributed to the observed variability in prevalence estimates and associations, as instruments may have captured overlapping but non-identical CF dimensions. Consequently, reported CF prevalence estimates should be interpreted as contextually and conceptually bounded rather than directly comparable across studies.

### Interventions

Interventions varied in focus and mechanism, including individual-level resilience and self-compassion training (targeting emotional regulation), peer-support programmes (social buffering), motivational SMS interventions (engagement and morale), and mobile psychoeducation (knowledge and coping skills), generally yielding small-to-moderate benefits [1,55–57]. Outcome measures differed substantially, limiting cross-intervention comparability [1,55–57]. These interventions differed substantially in theoretical orientation, target populations, delivery mode, and outcome measures, limiting direct comparison and synthesis of effectiveness across intervention types. Overall, evidence remains limited in scope, with few robust evaluations and inconsistent methods, underscoring the need for robust, context-sensitive evaluations across diverse healthcare settings and time periods.

### Synthesis of evidence on compassion fatigue

Across the 56 included studies, CF was consistently reported as a pervasive occupational hazard among healthcare workers, with prevalence estimates ranging widely from 20 to 87%. Moderate-to-high CF levels were most frequently observed among frontline nurses, intensive care and emergency staff, early-career professionals, and women. This wide prevalence range reflects substantial methodological and contextual heterogeneity rather than uniform burden, including differences in conceptual definitions of CF, measurement instruments and cut-off thresholds, sampling strategies, timing of data collection across pandemic phases, and variability in healthcare system capacity. Higher prevalence estimates were more commonly reported in studies using lower CF threshold scores, frontline or high-exposure samples, and data collected during early pandemic surges.

Vulnerability to CF emerged from the convergence of demographic, occupational, and systemic factors. Commonly identified demographic correlates included younger age, female gender, and fewer years of professional experience. Occupational contributors comprised high workload, extended or night shifts, repeated exposure to patient suffering and death, and moral distress [4,6,15,29]. Systemic and contextual stressors, particularly resource scarcity, rural or under-resourced practice environments, inadequate organisational support, and institutional value conflicts, were also frequently reported. Organisational and policy-level determinants were explicitly examined in 18 studies, most commonly through empirical measures of staffing adequacy, managerial support, workplace safety, and access to psychosocial services.

Symptom profiles associated with CF were relatively consistent across settings, with emotional exhaustion, depersonalisation, diminished empathy, and intrusive or distressing thoughts most frequently reported [1–7,12–44,46–49,51–60]. These symptoms often co-occurred with anxiety, depression, and secondary traumatic stress. Reported effects extended beyond individual psychological distress to include reduced compassion satisfaction, impaired work performance, increased self-reported clinical errors, and higher turnover intention [1–7,12–44,46–49,51–60]. Protective factors identified across studies included individual resilience, peer and social support, self-compassion, and spiritual well-being. Interventions targeting these domains, most commonly mindfulness-based approaches and psychoeducational strategies, demonstrated small-to-moderate benefits, although outcome measures and evaluation designs varied substantially [1–7,12–22,26–44,46–49,51,52,54–60].

Evidence on temporal trends was limited. Only 10 studies employed repeated cross-sectional or longitudinal designs [1,6,16,40,50,55–58]; therefore, observed peaks during early pandemic surges should be interpreted cautiously, as most evidence derives from cross-sectional snapshots rather than true temporal follow-up. Available evidence suggests that CF peaked during early pandemic surges, particularly among critical care teams, and declined modestly as workloads and organisational supports stabilised [1,6,16,40,50,55–58]. Yet, evidence from several contexts indicates persistent or delayed CF, especially in resource-limited or long-COVID settings [34–39,41,43]. While quantitative studies often reported moderate prevalence levels, qualitative findings revealed deeper psychological consequences, highlighting moral distress, compromised care quality, depression, and intent to leave employment [1,6,16,40,50,55–58].

## DISCUSSION

This review demonstrates that CF represented a significant and underappreciated occupational burden for HCWs during the COVID-19 pandemic, raising concerns for workforce retention, quality of care, and patient safety dur­ing public health emergencies. Drawing on evidence from 56 studies predominantly employing cross-sectional designs, nurses, women, and staff working in high-intensity or resource-limited environments were consistently identified as the most vulnerable groups. The wide range of reported prevalence estimates reflects not only elevated exposure to pandemic-related stressors but also heterogeneity in study design, measurement approaches, and timing of data collection [60]. Collectively, the evidence underscores CF as a multifactorial and context-dependent phenomenon, shaped by the interaction between individual resilience, workplace culture, and systemic response capacity, rather than as a uniform or static condition.

The current CF evidence base is constrained by important conceptual and methodological limitations. Although CF overlaps with burnout, moral distress, and secondary traumatic stress, it remains analytically distinct in its emphasis on relational exposure to patient suffering and sustained caregiving demands [61]. In the reviewed studies, this distinction was not always consistently operationalised, particularly in research relying on ProQOL subscales, which capture intersecting but non-identical occupational stress constructs [61]. Cross-sectional designs and heterogeneous measurement tools limit causal inference and impede assessment of temporal sequencing between risk factors, CF, and downstream outcomes. These constraints reinforce the need for longitudinal studies capable of capturing trajectories of CF over time and for rigorously evaluated interventions that can establish effectiveness beyond short-term or context-specific effects.

Another key challenge identified is the uneven global distribution of studies. Although the review identified recurring patterns, findings are disproportionately shaped by evidence from Asia and high-income Western health systems. This geographic concentration likely influences dominant conceptualisations of CF and the types of stressors most frequently examined, while potentially obscuring alternative expressions of CF in under-resourced or collectivist settings such as sub-Saharan Africa. Cultural norms surrounding emotional expression, caregiving obligations, and moral distress may influence both the experience and reporting of CF, suggesting that current estimates may underrepresent or mischaracterise CF in low-resource contexts [17]. Consequently, generalisation of findings to underrepresented regions should be approached with caution, and future research should prioritise context-sensitive designs that explicitly account for cultural, systemic, and resource-related variability.

Importantly, organisational and policy-level determinants of CF remain insufficiently examined relative to individual-level factors. While 18 studies empirically assessed organisational variables such as staffing adequacy, managerial support, workplace safety, and access to psychosocial services, the majority of interventions focused narrowly on individual coping strategies. Despite consistent associations between workload, staffing pressures, psychosocial support availability, and CF vulnerability [1,55,58,60], structural responses, such as institutional mental health services, staff redistribution, and violence prevention policies, were rarely evaluated as primary intervention targets remained underexplored, despite their likely positive impact on reducing CF [39,46,48,51,60]. This imbalance constrains the evidence base needed to inform system-level responses with sustained impact.

Collectively, these findings indicate that CF during COVID-19 should not be conceptualised solely as an individual psychological issue requiring an individual-level response, but rather as a systemic occupational health challenge embedded within organisational and policy environments [44,46,52,60]. Effective mitigation therefore requires coordinated, multi-level approaches that integrate individual-focused strategies with organisational reform and supportive policy frameworks [60]. Without addressing structural drivers alongside personal coping resources, efforts to protect HCW well-being and strengthen health system resilience are likely to remain fragmented and insufficient.

Finally, variation in the theoretical framing of CF across measurement instruments further complicates the synthesis and interpretation of the evidence. ProQOL conceptualises CF as a composite of burnout and secondary traumatic stress, whereas CF-SS emphasises emotional depletion arising from caregiving exposure [61,62]. These divergent conceptual foundations, combined with differences in scoring methods and cut-off thresholds across study contexts, mean that prevalence estimates may capture overlapping but distinct dimensions of occupational distress. As a result, observed variability in CF prevalence and associated factors should be interpreted as reflecting both contextual differences and instrument-specific constructs, rather than as directly comparable indicators of burden across studies.

### Study limitations

This scoping review has several limitations that should be considered when interpreting the findings. Comparability across studies was constrained by substantial heterogeneity in study designs, measurement tools, populations, and healthcare contexts. Variations in conceptual definitions of compassion fatigue, use of different instruments (*e.g.* ProQOL *vs*. CF-SS), and inconsistent cut-off thresholds likely contributed to the wide range of reported prevalence estimates and limit direct comparison or aggregation of results across settings.

The predominance of cross-sectional, self-report studies introduced important sources of bias. Reliance on self-reported measures may have inflated prevalence estimates due to recall bias and social desirability effects, particularly in high-stress pandemic contexts. Moreover, the cross-sectional nature of most studies precludes causal inference, limiting the ability to determine temporal relationships between risk factors, CF, and outcomes, as well as to draw firm conclusions regarding the effectiveness of reported interventions.

Geographic representation was uneven, with evidence disproportionately derived from Asia and high-income Western countries, and limited representation from sub-Saharan Africa and South America. This imbalance restricts the global applicability of the findings and may underrepresent the influence of culturally specific norms, systemic constraints, and resource-limited healthcare environments on the experience and reporting of CF.

The small number of rigorously designed interventional studies limits confidence in conclusions about effective mitigation strategies. Most interventions were evaluated using small samples, short follow-up periods, or pre-post designs without control groups, thereby constraining the strength of inferences regarding sustainability and real-world effectiveness. Collectively, these limitations underscore the need for more methodologically robust, longitudinal, and context-sensitive research to strengthen the evidence base on compassion fatigue among healthcare workers.

### Key recommendations

The evidence mapped in this review indicates that strengthening organisational and policy-level protections for healthcare workers is the most urgent and well-supported priority. Across diverse settings, CF was consistently associated with excessive workload, staffing shortages, moral distress, and limited institutional support, underscoring the need for systemic interventions such as workforce planning, access to psychosocial and peer-support services, workplace safety measures, and organisational policies that explicitly address occupational mental health risks.

Integrated multi-level strategies represent the most sustainable approach to mitigation, as individual-focused interventions, such as resilience, self-compassion, and mindfulness training, demonstrated modest benefits but were unlikely to be effective in isolation. Embedding these approaches within supportive organisational cultures and enabling policy environments is essential for durable impact on workforce well-being and retention.

Improving research quality and causal inference remains a critical priority, given the predominance of cross-sectional designs and heterogeneous measurement approaches. Longitudinal and rigorously designed interventional studies are needed to clarify temporal dynamics, evaluate intervention effectiveness, and distinguish pandemic-related distress from persistent compassion fatigue.

Expanding evidence from underrepresented regions is necessary to enhance global relevance and equity. The concentration of studies in high-income and middle-income settings limits the transferability of findings, highlighting the importance of context-sensitive research that accounts for cultural norms, health system capacity, and resource constraints in low-resource environments.

## CONCLUSIONS

The COVID-19 pandemic revealed compassion fatigue as a critical occupational hazard, disproportionately affecting nurses, women, and frontline healthcare staff. Evidence gaps, methodological limitations, and a predominant focus on individual coping over systemic solutions suggest that CF is under-prioritised as a structural workforce challenge, requiring more attention in future research and targeted interventions. Addressing it requires longitudinal research, global representation, and multi-level strategies that combine resilience, organisational reform, and policy action to safeguard healthcare worker well-being in future crises.

## References

[1] Goktas S Gezginci E Kartal H The effects of motivational messages sent to emergency nurses during the COVID-19 pandemic on job satisfaction, compassion fatigue, and communication skills: a randomized controlled trial. J Emerg Nurs. 2022;48:547–558. 10.1016/j.jen.2022.06.001 35864005 PMC9226325

[2] Christianson J Johnson N Nelson A Singh M Work-related burnout, compassion fatigue, and nurse intention to leave the profession during COVID-19. Nurse Lead. 2023;21:244–251. 10.1016/j.mnl.2022.06.007 35783544 PMC9239979

[3] Labrague LJ de Los Santos JAA Resilience as a mediator between compassion fatigue, nurses’ work outcomes, and quality of care during the COVID-19 pandemic. Appl Nurs Res. 2021;61:151476. 10.1016/j.apnr.2021.151476 34544570 PMC8448586

[4] Arıkan A Esenay FI Compassion fatigue and burnout in Turkish pediatric emergency nurses during the COVID-19 pandemic. J Pediatr Nurs. 2023;71:120–126. 10.1016/j.pedn.2022.11.004 36424330 PMC9678743

[5] Munjappa HB Parekh MK Patil AN Shinde SA Ingale SS Munjappa H The Unseen Toll: Impact of COVID-19 on Professional Quality of Life of Healthcare Workers. Cureus. 2025;17:e88633. 10.7759/cureus.88633 40861597 PMC12374776

[6] Vracevic M Pavlovic V Todorovic N Milic NM Matejic B Brkic P Compassion fatigue and satisfaction among frontline staff in long term care facilities: psychometric properties of the Serbian version of the professional quality of life scale. Front Psychiatry. 2025;16:1479190. 10.3389/fpsyt.2025.1479190 40130190 PMC11931146

[7] Poudyal S Sharma K Ghimire S Compassion fatigue, burnout and compassion satisfaction among nurses working in a tertiary care hospital of Nepal during COVID-19 pandemic. Journal of Chitwan Medical College. 2022;12:19–22. 10.54530/jcmc.1070

[8] Pollock D Tricco AC Peters MD Mclnerney PA Khalil H Godfrey CM Methodological quality, guidance, and tools in scoping reviews: a scoping review protocol. JBI Evid Synth. 2022;20:1098–1105. 10.11124/JBIES-20-00570 34446668

[9] McGowan J Straus S Moher D Langlois EV O’Brien KK Horsley T Reporting scoping reviews—PRISMA ScR extension. J Clin Epidemiol. 2020;123:177–179. 10.1016/j.jclinepi.2020.03.016 32229248

[10] Braun V Clarke V Using thematic analysis in psychology. Qual Res Psychol. 2006;3:77–101. 10.1191/1478088706qp063oa

[11] Wong WC Cheung CS Hart GJ Development of a quality assessment tool for systematic reviews of observational studies (QATSO) of HIV prevalence in men having sex with men and associated risk behaviours. Emerg Themes Epidemiol. 2008;5:23. 10.1186/1742-7622-5-23 19014686 PMC2603000

[12] Aydin Dogan R Huseyinoglu S Yazici S Compassion fatigue and moral sensitivity in midwives in COVID-19. Nurs Ethics. 2023;30:776–788. 10.1177/09697330221146224 36927231 PMC10028444

[13] Taş F Aşcı Ö Bal MD Compassion fatigue and satisfaction in nurses and midwives during the COVID-19 pandemic in Turkey. Clinical and Experimental Health Sciences. 2022;12:521–527. 10.33808/clinexphealthsci.998790

[14] Karaca A Emül E The effect of compassion fatique on work satisfaction in healthcare professionals during the covid-19 pandemic period. Sosyal Araştırmalar ve Yönetim Dergisi. 2022:89–104. 10.35375/sayod.1073836

[15] Uslu E Kendirkıran G Compassion fatigue and risk factors in nurses in the covid-19 pandemic. Dokuz Eylül Üniv Hemsire Fak Elektron Derg. 2022;15:298–306. 10.46483/deuhfed.1010957

[16] Çakmak MA İlhan T A Study on Compassion Fatigue in Health Workers During the COVID-19 Pandemic. Uluslararası Türk Eğitim Bilimleri Dergisi. 2024;12:584–612. 10.46778/goputeb.1325100

[17] Keskin AY Molu B Teke ZB The effect of COVID-19 pandemic period upon nurses’ compassion fatigue. Sağlık Akademisyenleri Dergisi. 2024;11:237–245.

[18] Hochwarter W Jordan S Kiewitz C Liborius P Lampaki A Franczak J Losing compassion for patients? The implications of COVID-19 on compassion fatigue and event-related post-traumatic stress disorder in nurses. J Manag Psychol. 2022;37:206–223. 10.1108/JMP-01-2021-0037

[19] Abou Hashish EA Ghanem Atalla AD The relationship between coping strategies, compassion satisfaction, and compassion fatigue during the COVID-19 pandemic. SAGE Open Nurs. 2023;9:23779608231160463. 10.1177/23779608231160463 36908330 PMC9998409

[20] Dwyer ML Alt M Brooks JV Katz H Poje AB Burnout and compassion satisfaction: survey findings of healthcare employee wellness during COVID-19 pandemic using ProQOL. Kans J Med. 2021;14:121. 10.17161/kjm.vol1415171 34084270 PMC8158419

[21] De Los Santos JAA Compassion fatigue influences the mental health and turnover intention of nurses in the COVID-19 pandemic. Acta Med Philipp. 2023;57:19. 39484195 10.47895/amp.vi0.5137 PMC11522638

[22] Smart D Glubrecht A Brooks O Graves J COVID-19 Impact on Compassion Fatigue and Career Decisions among Registered Nurses. Creat Educ. 2024;15:941–957. 10.4236/ce.2024.155057

[23] Ariapooran S Abdolomaleki B Compassion fatigue in nurses: the role of spiritual well-being, emotion regulation and time perspective. Iran J Nurs Midwifery Res. 2023;28:150–154. 10.4103/ijnmr.ijnmr_293_21 37332382 PMC10275470

[24] Dilapdilap R Marzan P Compassion Fatigue and Psychological Well-Being of Nurses as Moderated by Spiritual Orientation Amidst Covid-19 Pandemic: A Basis for Spiritual Wellness Program Module. Psychology and Education: A Multidisciplinary Journal. 2023;9:1–11.

[25] Minò MV Vacca A Colizzi I Solomita B Franza F Tavormina G The effect of the pandemic on the care of patients with mental disorders: measure of” compassion fatigue” and” burn-out” in the operator. Psychiatr Danub. 2021;33:114–118. 34559789

[26] Tamuno-opubo AT Uthman JT Idehen EE Baba KA Examining the Moderating Role of Self-Compassion in the Relationship Between Psychache, Compassion Fatigue, and General Well-Being among Nurses in the Aftermath of COVID-19. Journal of Client-Centered Nursing Care. 2024;10:249–261.

[27] Hăisan A Hogaș S Măirean C Punei MO Volovăț SR Hogaș M Compassion fatigue and compassion satisfaction among Romanian emergency medicine personnel. Front Med (Lausanne). 2023;10:1189294. 10.3389/fmed.2023.1189294 37554501 PMC10406243

[28] Zakeri MA Rahiminezhad E Salehi F Ganjeh H Dehghan M Compassion satisfaction, compassion fatigue and hardiness among nurses: a comparison before and during the COVID-19 outbreak. Front Psychol. 2022;12:815180. 10.3389/fpsyg.2021.815180 35222165 PMC8866727

[29] Yi LJ Cai J Ma L Lin H Yang J Tian X Prevalence of Compassion Fatigue and its Association with Professional Identity in Junior College nursing interns: a cross-sectional study. Int J Environ Res Public Health. 2022;19:15206. 10.3390/ijerph192215206 36429923 PMC9690934

[30] Ariapooran S Amirimanesh M Turnover intention of nurses in the outbreak of COVID-19: the role of compassion fatigue, compassion satisfaction and burnout. Quarterly Journal of Nursing Management. 2021;10:80–93.

[31] Zhan Y Liu Y Chen Y Liu H Zhang W Yan R The prevalence and influencing factors for compassion fatigue among nurses in Fangcang shelter hospitals: A cross-sectional study. Int J Nurs Pract. 2022;28:e13054. 10.1111/ijn.13054 35384160

[32] Xia W Defang W Xiaoli G Jinrui C Weidi W Junya L Compassion satisfaction and compassion fatigue in frontline nurses during the COVID-19 pandemic in Wuhan, China. J Nurs Manag. 2022;30:2537–2548. 10.1111/jonm.13777 36042535 PMC9538334

[33] Ruiz-Fernández MD Ramos-Pichardo JD Ibáñez-Masero O Cabrera-Troya J Carmona-Rega MI Ortega-Galán ÁM Compassion fatigue, burnout, compassion satisfaction and perceived stress in healthcare professionals during the COVID-19 health crisis in Spain. J Clin Nurs. 2020;29:4321–4330. 10.1111/jocn.15469 32860287

[34] Zeng L Liu D Liang X Li L Peng Y Jin M The levels and influencing factors of compassion fatigue among new nurses during the COVID-19 pandemic. J Nurs Manag. 2023;2023:4362841. 10.1155/2023/4362841 40225694 PMC11919100

[35] Omri N Ezzi O Ammar A Benzarti W Loghmari D Toulgui E Compassion fatigue among frontline healthcare workers during the covid-19 pandemic in Tunisia. PLoS One. 2022;17:e0276455. 10.1371/journal.pone.0276455 36301952 PMC9612510

[36] Fernández-Miranda G Urriago-Rayo J Akle V Noguera E Mejía N Amaya S Compassion and decision fatigue among healthcare workers during COVID-19 pandemic in a Colombian sample. PLoS One. 2023;18:e0282949. 10.1371/journal.pone.0282949 36961780 PMC10038311

[37] Kachie ADT Zhou L Quansah PE Xu X Epalle TM Ngajie BN Role demands and turnover intention among Covid-19 frontline nurses: The mediating and moderating roles of compassion fatigue and spiritual leadership. PLoS One. 2023;18:e0289888. 10.1371/journal.pone.0289888 37561736 PMC10414576

[38] Bailey BC Cox S Terris L Van Oppen D Howsare J Berry JH Rural health care worker wellness during COVID-19: Compassion fatigue, compassion satisfaction & utilization of wellness resources. PLoS One. 2023;18:e0295020. 10.1371/journal.pone.0295020 38064476 PMC10707602

[39] De Luca R Bonanno M Maggio MG Todaro A Rifici C Mento C Compassion Fatigue in a Cohort of South Italian Nurses and Hospital-Based Clinical Social Workers Following COVID-19: A Cross-Sectional Survey. J Clin Med. 2024;13:4200. 10.3390/jcm13144200 39064240 PMC11278230

[40] Fahmy AM Saber EH Gabra SF Relation between Compassion Fatigue, Pandemic Emotional Impact, and Time Management among Nurses at Isolation Hospitals during COVID-19. Minia Scientific Nursing Journal. 2022;12:57–68. 10.21608/msnj.2022.163882.1037

[41] Amir K Okalo P Frontline nurses’ compassion fatigue and associated predictive factors during the second wave of COVID-19 in Kampala, Uganda. Nurs Open. 2022;9:2390–2396. 10.1002/nop2.1253 35633514 PMC9348371

[42] Taskiran Eskici G Uysal Kasap E Gumus E Relationships between leadership behaviour of nurse managers and nurses’ levels of job satisfaction and compassion fatigue during the COVID-19 pandemic. Nurs Open. 2023;10:4548–4559. 10.1002/nop2.1701 36879354 PMC10277410

[43] Uhlig-Reche H Rolin S Karnik R Lyons M Verduzco-Gutierrez M Compassion fatigue, work engagement, and psychological distress in health care workers treating patients with long COVID. PM R. 2025;17:1162–1169. 10.1002/pmrj.13383 40249096 PMC12354227

[44] Akanni AA Ajele KW Oduaran CA Moderating effect of resilience in the relationship between compassion fatigue and mental well-being among frontline health workers exposed to COVID-19 Patients. Rom J Appl Psychol. 2023;25:9–13.

[45] Alreshidi SM Rayani AM Predictors of compassion satisfaction, compassion fatigue, and burnout among nursing professionals in a medical City in Saudi Arabia. Risk Manag Healthc Policy. 2023;16:2883–2892. 10.2147/RMHP.S430082 38149179 PMC10750782

[46] Rahman A Duan Y Symonds-Brown H Salma J Estabrooks CA Care Aides Compassion Fatigue, Burnout, and Compassion Satisfaction Related to Long-Term Care (LTC) Working Environment. J Appl Gerontol. 2026;45:3–16. 10.1177/07334648251328400 40126450 PMC12681364

[47] Sahin S Arioz Duzgun A Unsal A Inan Kirmizigul E Ozdemir A Assessment of compassion fatigue and empathy levels in nurses during the COVID-19 outbreak: Turkey’s case. J Relig Health. 2023;62:1343–1357. 10.1007/s10943-023-01749-z 36719601 PMC9888325

[48] Li JN Jiang XM Zheng QX Lin F Chen XQ Pan YQ Mediating effect of resilience between social support and compassion fatigue among intern nursing and midwifery students during COVID-19: a cross-sectional study. BMC Nurs. 2023;22:42. 10.1186/s12912-023-01185-0 36788572 PMC9928591

[49] Liu D Xie S Jing J Niyomsilp E Xie L Nie X The effect of perceived organizational support and ego-resilience on the relationship between occupational stressors and compassion fatigue in COVID-19 frontline nurses: a cross-sectional study in Sichuan, China. BMC Nurs. 2024;23:817. 10.1186/s12912-024-02473-z 39529080 PMC11556187

[50] Jafarian Amiri SR Qalehsari MQ Zabihi A Babanataj R Chehrazi M The relationship between empowerment and compassion satisfaction, compassion fatigue, and burnout in nurses during COVID-19 outbreak. J Educ Health Promot. 2023;12:379. 38144007 10.4103/jehp.jehp_504_23 PMC10743936

[51] Cosentino C Foà C Bertuol M Cappi V Riboni S Rossi S The impact of the alterations in caring for COVID-19 patients on Compassion Satisfaction and Compassion Fatigue in Italian nurses: a multi method study. Acta Biomed. 2022;93:e2022190. 35545974 10.23750/abm.v93iS2.13053 PMC9534219

[52] Chatham AA Petruzzi LJ Patel S Brode WM Cook R Garza B Structural Factors Contributing to Compassion Fatigue, Burnout, and Secondary Traumatic Stress Among Hospital-Based Healthcare Professionals During the COVID-19 Pandemic. Qual Health Res. 2024;34:362–373. 10.1177/10497323231213825 38011747 PMC10905984

[53] Dearborn R. Women healthcare workers, compassion fatigue, and the COVID-19 pandemic [master’s thesis]. Winnipeg, Manitoba, Canada: University of Manitoba; 2024.

[54] Missouridou E Mangoulia P Pavlou V Kritsotakis E Stefanou E Bibou P Wounded healers during the COVID-19 syndemic: Compassion fatigue and compassion satisfaction among nursing care providers in Greece. Perspect Psychiatr Care. 2022;58:1421–1432. 10.1111/ppc.12946 34505638 PMC8661928

[55] Koller EC Abel RA Milton LE Caring for the caregiver: A feasibility study of an online program that addresses compassion fatigue, burnout, and secondary trauma. Open J Occup Ther. 2022;10:1–14. 10.15453/2168-6408.1847

[56] Sayadi I Moradian ST Mahmoudi H Salimi SH Moayed MS Effectiveness of self-compassion on compassion fatigue and resilience of nurses in intensive care unit for COVID-19. Curr Psychol. 2025;44:7612–7619. 10.1007/s12144-025-07277-1

[57] McCool N Reidy J Steadman S Nagpal V The buddy system: an intervention to reduce distress and compassion fatigue and promote resilience on a palliative care team during the COVID-19 pandemic. J Soc Work End Life Palliat Care. 2022;18:302–324. 10.1080/15524256.2022.2122650 36129825

[58] Gür S Katran HB Arpag N Öztekin D Perceptions and Experiences of Critical Care Nurses on Compassion Fatigue in the COVID-19 Pandemic: A Qualitative Study. Hemşirelikte Araştırma Geliştirme Dergisi. 2023;25:47–60.

[59] Vacca A Minò MV Longo R Lucisani G Solomita B Franza F The emotional impact on mental health workers in the care of patients with mental disorders in the pandemic and post COVID-19 pandemic: a measure of ‘Burnout’and ’Compassion Fatigue’. Psychiatr Danub. 2023;35:292–295. 37800243

[60] Jo S Kurt Ş Mayer K Pituch KA Simpson V Skibiski J Compassion fatigue and COVID-19: A global view from nurses. Worldviews Evid Based Nurs. 2023;20:116–125. 10.1111/wvn.12641 37026170

[61] Kaya E Molu NG Relationship between moral distress, compassion fatigue, and burnout levels of psychiatric nurses. J Psychiatr Nurs. 2023;14:103.

[62] Slusarz C Understanding the Experiences of Medical Interpreters in Palliative Care: An Exploration of Measurement Tools. Illn Crises Loss. 2024;32:446–460. 10.1177/10541373241231441

